# Iron–sulfur cluster assembly scaffold protein IscU is required for activation of ferric uptake regulator (Fur) in *Escherichiacoli*

**DOI:** 10.1016/j.jbc.2024.107142

**Published:** 2024-03-05

**Authors:** Aidan G. Purcell, Chelsey R. Fontenot, Huangen Ding

**Affiliations:** Department of Biological Sciences, Louisiana State University, Baton Rouge, Louisiana, USA

**Keywords:** iron homeostasis, iron–sulfur cluster biogenesis, ferric uptake regulator (Fur)

## Abstract

It was generally postulated that when intracellular free iron content is elevated in bacteria, the ferric uptake regulator (Fur) binds its corepressor a mononuclear ferrous iron to regulate intracellular iron homeostasis. However, the proposed iron-bound Fur had not been identified in any bacteria. In previous studies, we have demonstrated that *Escherichia coli* Fur binds a [2Fe-2S] cluster in response to elevation of intracellular free iron content and that binding of the [2Fe-2S] cluster turns on Fur as an active repressor to bind a specific DNA sequence known as the Fur-box. Here we find that the iron–sulfur cluster assembly scaffold protein IscU is required for the [2Fe-2S] cluster assembly in Fur, as deletion of IscU inhibits the [2Fe-2S] cluster assembly in Fur and prevents activation of Fur as a repressor in *E. coli* cells in response to elevation of intracellular free iron content. Additional studies reveal that IscU promotes the [2Fe-2S] cluster assembly in apo-form Fur and restores its Fur-box binding activity *in vitro*. While IscU is also required for the [2Fe-2S] cluster assembly in the *Haemophilus influenzae* Fur in *E. coli* cells, deletion of IscU does not significantly affect the [2Fe-2S] cluster assembly in the *E. coli* ferredoxin and siderophore-reductase FhuF. Our results suggest that IscU may have a unique role for the [2Fe-2S] cluster assembly in Fur and that regulation of intracellular iron homeostasis is closely coupled with iron–sulfur cluster biogenesis in *E. coli*.

The ferric uptake regulator (Fur) is the founding member of the Fur superfamily that regulates intracellular metal homeostasis in bacteria (1, 2). In *Escherichia coli*, Fur regulates over 158 genes (3, 4, 5) involved in intracellular iron homeostasis, energy metabolism, DNA synthesis, oxidative stress response, and bacterial virulence (6, 7). In the past 3 decades, it was postulated that when intracellular free iron content is elevated, Fur binds its corepressor a mononuclear ferrous iron (1, 2, 8, 9) and regulates the expression of its target genes by binding to a specific DNA sequence known as the Fur-box (10, 11, 12). However, there is no concrete evidence supporting the idea that Fur binds ferrous iron in *E. coli* or any other bacteria. A number of Fur crystal structures including *E. coli* Fur (13), *Mycobacterium tuberculosis* Fur (14), *Vibrio cholerae* Fur (15), *Helicobacter pylori* Fur (16), *Campylobacter jejuni* Fur (17), and *Francisella tularensis* Fur (18) have been reported, and none of these structures contains iron. Fur proteins exist as a dimer or tetramer (19), and each Fur monomer has three putative metal binding sites (13, 15): site 1 is located within the dimerization domain; site 2 connects the DNA binding domain and the dimerization domain, and site 3 is positioned at the C-terminal end of the dimerization domain. In purified Fur proteins, site 1 and site 2 are often occupied by zinc (15, 20). Interestingly, the *in vitro* studies have showed that *E. coli* Fur has a relatively weak binding activity for ferrous iron with dissociation constants ranging from 1.2 μM to 55 μM (21, 22). However, since the intracellular free iron concentration in *E. coli* cells grown in LB medium is estimated to be less than 1 μM (23), it may explain why Fur does not bind any ferrous iron in *E. coli* cells.

Serendipitously, we have found that *E. coli* Fur binds a [2Fe-2S] cluster in *E. coli* cells in which intracellular free iron content is elevated (24, 25, 26). A suite of biophysical techniques including the electron paramagnetic resonance and Mössbauer spectroscopy have been used to assign a [2Fe-2S] cluster binding in Fur in *E. coli* cells in response to elevation of intracellular iron content (25). Additional studies have revealed that binding of the [2Fe-2S] cluster in Fur turns on its binding activity for the Fur-box (24). Furthermore, binding of the [2Fe-2S] cluster in Fur is highly conserved, as Fur proteins from *Haemophilus influenzae*, *V. cholerae*, and *H. pylori* also bind a [2Fe-2S] cluster in *E. coli* cells with an elevated intracellular free iron content (27). The results suggest that *E. coli* Fur binds a [2Fe-2S] cluster in response to elevation of intracellular free iron content and regulates intracellular iron homeostasis (24, 25, 26).

In *E. coli* and many other bacteria, iron–sulfur clusters in proteins are assembled by two iron–sulfur cluster assembly systems: the housekeeping *isc* operon (*iscSUA-hscBA-fdx*) (28) and the alternative *suf* operon (*sufABCDSE*) (29, 30). While the expression of the *suf* operon is induced under iron starvation (30) or oxidative stress (31, 32) conditions, the housekeeping *isc* operon is mainly responsible for iron–sulfur cluster assembly in cells under normal growth conditions (28). Among the core iron–sulfur cluster assembly proteins encoded by the housekeeping *isc* operon, IscS is a cysteine desulfurase that catalyzes desulfurization of L-cysteine and delivers sulfur for iron–sulfur cluster assembly in IscU (33, 34, 35, 36). IscU is a scaffold protein that assembles iron–sulfur clusters and transfers the assembled clusters to target proteins (37, 38, 39, 40). IscA was initially characterized as an alternative scaffold protein (41). However, unlike IscU, IscA has a very strong iron binding activity and may act as an iron chaperone for the [4Fe-4S] cluster assembly in proteins (42, 43, 44, 45, 46). Previously, we have found that deletion of IscA and its paralog SufA elevates intracellular free iron content and promotes the [2Fe-2S] cluster assembly in Fur in *E. coli* cells (25), indicating that IscA is not directly involved in the [2Fe-2S] cluster assembly in Fur.

Here we report that deletion of the iron–sulfur cluster assembly scaffold protein IscU inhibits the [2Fe-2S] cluster assembly in Fur and prevents activation of Fur as a repressor in *E. coli* cells in response to elevation of intracellular free iron content. The *in vitro* studies further show that IscU promotes the [2Fe-2S] cluster assembly in apo-form Fur and restores its Fur-box binding activity. While IscU is also required for the [2Fe-2S] cluster assembly in the *H. influenzae* Fur expressed in *E. coli* cells, deletion of IscU has very little or no effects on the [2Fe-2S] cluster assembly in the *E. coli* ferredoxin (47, 48) and siderophore reductase FhuF (49, 50), indicating that IscU may have a unique role for the [2Fe-2S] cluster assembly in Fur. The interplay between regulation of intracellular iron homeostasis and iron–sulfur cluster biogenesis in *E. coli* cells will be discussed.

## Results

### The [2Fe-2S] cluster assembly in the Fur requires the iron–sulfur cluster assembly scaffold IscU in *E. coli* cells

**Figure 1:**
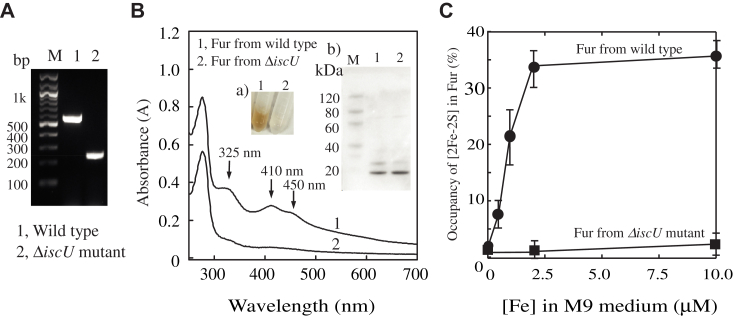
**The iron–sulfur cluster assembly scaffold protein IscU is required for the [2Fe-2S] cluster assembly in Fur in *E. coli* cells.***A*, PCR confirmation of gene *iscU* deletion in *E. coli* cells. Gene *iscU* was deleted using the single-step inactivation procedure (52) as described previously (43). Two primers (primer 1, 5′-TGACCTTTCTCCGCTGTGGG-3′, and primer 2, 5′-CTTTACCGCGGTTAGCCAGA-3′) were used for the PCR confirmation of the gene *iscU* deletion. Lane M, 100 bp DNA ladder (NEB). Lane 1, from wildtype *E. coli* cells. Lane 2, from the Δ*iscU* mutant cells. *B*, UV-Vis absorption spectra of Fur proteins purified from wildtype *E. coli* cells (spectrum 1) and the Δ*iscU* mutant cells (spectrum 2) grown in M9 medium supplemented with Fe(NH_4_)_2_(SO_4_)_2_ (2 μM) under aerobic growth conditions. Purified Fur proteins (50 μM) were dissolved in buffer containing NaCl (500 mM) and Tris (20 mM, pH 8.0). Insert (a), the photograph of purified Fur from wildtype *E. coli* cells (1) and the Δ*iscU* mutant cells (2). Insert (b), the SDS-PAGE gel of Fur proteins purified from wildtype *E. coli* cells (lane 1) and the Δ*iscU* mutant cells (lane 2) grown in M9 medium supplemented with Fe(NH_4_)_2_(SO_4_)_2_ (2.0 μM) (lane 2). Lane M, the PAGE-MASTER protein markers (GenScript co.) with molecular weights. *C*, the [2Fe-2S] cluster binding of Fur in *E. coli* wildtype and the Δ*iscU* mutant cells grown in M9 medium supplemented with increasing concentrations of iron. Fur protein was purified from *E. coli* wildtype and the Δ*iscU* mutant cells grown in M9 medium supplemented with indicated concentrations of Fe(NH_4_)_2_(SO_4_)_2_. The concentration of the [2Fe-2S] cluster in Fur was determined using an extinction coefficient of 10 mM^−1^ cm^−1^ at 410 nm (25). The [2Fe-2S] cluster occupancies of Fur were calculated from the ratio of the [2Fe-2S] cluster to Fur and plotted as a function of iron concentrations in M9 medium. Data represent mean ± SD (standard deviation) from three independent experiments. Fur, ferric uptake regulator.

To test whether the [2Fe-2S] cluster in Fur is assembled by the *isc* operon in *E. coli* cells, we have focused on the core iron–sulfur cluster assembly proteins: IscS, IscU, and IscA. IscS is required not only for iron–sulfur cluster biogenesis (33, 34, 35) but also for other key physiological processes such as tRNA modification (34) and biosynthesis of thiamine and NAD (51). Thus, deletion of IscS will affect multiple cellular functions (33). IscA is apparently not required for the [2Fe-2S] cluster assembly in Fur because deletion of IscA and its paralog SufA promotes the [2Fe-2S] cluster assembly in Fur in *E. coli* cells grown in LB medium (25). Therefore, we have constructed an *E. coli* mutant in which gene *iscU* is in-frame deleted using the single-step gene inactivation procedure (52). Deletion of gene *iscU* was confirmed by PCR (Fig. 1*A*). As reported previously by other groups (53, 54), deletion of IscU has only a mild effect on cell growth of *E. coli* in LB or M9 medium.

The plasmid encoding *E. coli* Fur (25) is then introduced into *E. coli* wildtype and the Δ*iscU* mutant cells. Both cells are grown in M9 medium supplemented with 2 μM iron (ferrous ammonium sulfate) under aerobic growth conditions (24). Figure 1*B* shows that the *E. coli* Fur purified from wildtype *E. coli* cells has a red color ((insert a) in Fig. 1*B*) and three distinct absorption peaks at 325 nm, 410 nm, and 450 nm of the [2Fe-2S] cluster (spectrum 1), as reported previously (24). In contrast, the *E. coli* Fur purified from the Δ*iscU* mutant cells grown in M9 medium supplemented with 2 μM iron has no color and very small absorption peaks at 325 nm, 410 nm, and 450 nm (Fig. 1*B*, spectrum 2). The iron and sulfur content analyses of the purified Fur proteins confirmed that Fur purified from the Δ*iscU* mutant cells contains very little or no iron–sulfur clusters. As reported previously, increase of the iron concentration in M9 medium to 10 μM does not increase the [2Fe-2S] cluster binding in the Fur in wildtype *E. coli* cells (Fig. 1*C*), indicating that 2 μM iron in M9 medium is sufficient to saturate the [2Fe-2S] cluster binding in Fur in wildtype *E. coli* cells (24). In parallel experiments, Fur expressed in the Δ*iscU* mutant cells remains as apo-form even when M9 medium is supplemented with 10 μM iron (Fig. 1*C*). Thus, IscU is essential for the [2Fe-2S] cluster assembly in Fur in *E. coli* cells in response to elevation of intracellular free iron content.

### Fur purified from the *E. coli* Δ*iscU* mutant cells fails to bind the Fur-box

Upon binding a [2Fe-2S] cluster, *E. coli* Fur becomes active to bind a specific DNA sequence known as the Fur-box (24). To explore the Fur-box binding activity of Fur purified from the *E. coli* Δ*iscU* mutant cells, we utilized the restriction site protection assay as described previously (24). The promoter region of the operon *iucABCD* which encodes the enzymes for biosynthesis of siderophore aerobactin has a consensus Fur-box sequence (5′-GAGAATCATTAGCATTCGC-3′) that also contains the restriction *hin*fI site (5′-GANTC-3′) (9). The promoter region of the operon *iucABCD* was cloned into plasmid pUC19 to create pUC19-*iuc* as described previously (24). Binding of Fur to the Fur-box protects the *hin*fI site from being cleaved by *Hin*fI (9) and creates a new DNA band of 787 bp (24).

**Figure 2:**
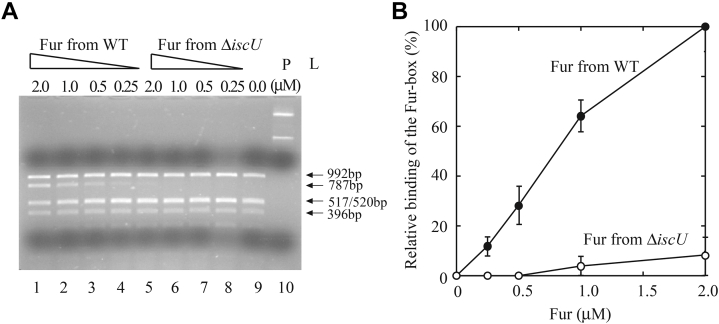
**The Fur-box binding activity of Fur purified from *E. coli* wildtype and the Δ*iscU* mutant cells grown in M9 medium supplemented with 2 μM iron.***A*, the restriction site protection assays of Fur. pUC19-*iuc* (3.2 nM) was preincubated with increasing concentrations of purified Fur proteins, followed by digestion with *Hin*fI (0.5 unit) at 37 ^°^C for 10 min. The digested DNA products were separated by 1.5% agarose gel electrophoresis. Lanes 1 to 4, pUC19-*iuc* (3.2 nM) was preincubated with 2.0 μM, 1.0 μM, 0.5 μM, and 0.25 μM Fur purified from wildtype *E. coli* cells, respectively. Lanes 5 to 8, pUC19-*iuc* (3.2 nM) was preincubated with 2.0 μM, 1.0 μM, 0.5 μM, and 0.25 μM Fur purified from the Δ*iscU* mutant *E. coli* cells, respectively. Lane 9, no Fur protein was added before the *Hin*fI digestion. Lane 10, pUC19-*iuc* only with no *Hin*fI digestion. *B*, relative binding activity of Fur proteins purified from wildtype and the Δ*iscU* mutant *E. coli* cells. The intensities of the DNA band of 787 bp shown in (*A*) were quantified using ImageJ and plotted as a function of the Fur concentrations. Data represent mean ± SD (standard deviation) from three independent experiments. Fur, ferric uptake regulator.

For the restriction site protection assays, pUC19-*iuc* is preincubated with increasing concentrations of Fur purified from wildtype *E. coli* cells, followed by the *Hin*fI digestion. As shown in Figure 2*A*, the *hin*fI restriction site of the Fur-box site is protected by the Fur purified from wildtype *E. coli* cells. In contrast, when pUC19-*iuc* is preincubated with increasing concentrations of the Fur purified from the *E. coli* Δ*iscU* mutant cells, followed by the *Hin*fI digestion, very little or no protection of the *hin*fI restriction site is observed (Fig. 2*A*). The intensities of the DNA band of 787 bp on the agarose gel are quantified using ImageJ (NIH) and plotted as a function of the Fur concentrations (Fig. 2*B*). The results demonstrate that Fur purified from the *E. coli* Δ*iscU* mutant cells lost its the Fur-box binding activity.

### Fur fails to repress its target gene in the *E. coli* Δ*iscU* mutant cells in response to elevation of intracellular free iron content

**Figure 3:**
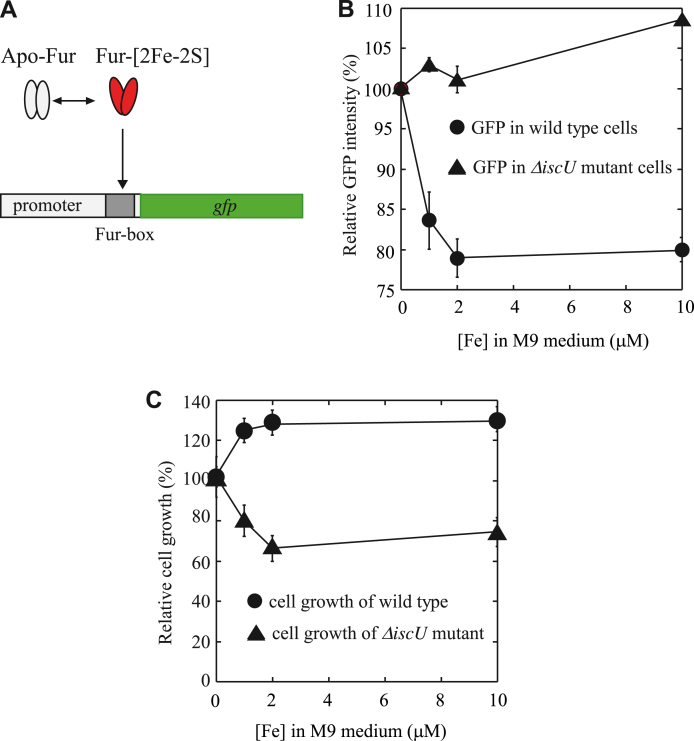
**Deletion of gene *iscU* prevents activation of Fur as a repressor in *E. coli* cells.***A*, a reporter gene *fur::gfp* was constructed and cloned into a plasmid pBAD as described in the Experimental Procedures. An active Fur represses the expression of the reporter gene *fur::gfp* and decreases the GFP fluorescence in *E. coli* cells. *B*, the repressor activity of Fur in *E. coli* wildtype and the *ΔiscU* mutant cells. The constructed plasmid containing the reporter gene *fur::gfp* was introduced into wildtype and the Δ*iscU* mutant cells. Wildtype *E. coli* and the *ΔiscU* mutant cells were grown in M9 medium supplemented with 0, 1.0 μM, 2.0 μM, and 10.0 μM Fe(NH_4_)_2_(SO_4_)_2._ After 5 h growth at 37 ^o^C under aerobic conditions, the cells were subjected to the GFP fluorescence measurements. The relative GFP intensities were obtained from the ratios of the GFP fluorescence intensity to the cell density (*A* at 600 nm) of the culture. The *E. coli* cells without the reporter gene *fur::gfp* were used as the controls. The data represent mean ± SD (standard deviation) from three independent experiments. *C*, deletion of gene *iscU* increases the sensitivity of *E. coli* cells to iron in M9 medium. Wildtype *E. coli* and the *ΔiscU* mutant cells were grown in M9 medium supplemented with 0, 1.0 μM, 2.0 μM, and 10.0 μM Fe(NH_4_)_2_(SO_4_)_2._ After 5 h growth at 37 ^°^C under aerobic conditions, the cell growth was measured at 600 nm. The ratios of the cell growth in M9 medium supplemented with increasing iron concentrations to the cell growth in M9 medium with no iron addition were plotted as a function of the iron concentrations in M9 medium. The *A* values at 600 nm (cell growth) of wildtype and the *ΔiscU* mutant grown in M9 medium with no iron addition were 0.74 ± 0.03 and 0.66 ± 0.04 for, respectively. The data represent mean ± SD (standard deviation) from three independent experiments. Fur, ferric uptake regulator; GFP, green fluorescent protein.

If deletion of IscU inhibits the [2Fe-2S] cluster assembly in Fur in *E. coli* cells (Fig. 1) and produces apo-form Fur that does not bind the Fur-box (Fig. 2), we expect that Fur may not be able to repress its target genes in the *E. coli* Δ*iscU* mutant cells in response to elevation of intracellular free iron content. To test this idea, we constructed a reporter gene *fur::gfp* encoding GFP (green fluorescent protein) controlled by a Fur-repressible promoter and inserted the reporter gene into plasmid pBAD (Fig. 3*A*), as described in Experimental Procedures. The constructed plasmid is introduced into *E. coli* wildtype and the Δ*iscU* mutant cells. The cells are grown in M9 medium supplemented with 0, 1.0 μM, 2.0 μM, and 10.0 μM iron, respectively. After 5 h of cell growth at 37 ^°^C under aerobic growth conditions, the cells are subjected to the GFP fluorescence measurements at 507 nm using the excitation wavelength at 481 nm (55). Relative GFP fluorescence intensities are calculated from the ratios of the fluorescence intensity to the cell density at 600 nm. Figure 3*B* shows that the expression of the reporter gene *fur::gfp* is repressed in wildtype *E. coli* cells grown in M9 medium supplemented with increasing concentrations of iron. About 2 μM iron in M9 medium is sufficient to repress the expression of the reporter gene *fur::gfp* (Fig. 3*B*), in agreement with the [2Fe-2S] cluster binding in Fur in wildtype *E. coli* cells under the same growth conditions (Fig. 1*C*). In contrast, the expression of the reporter gene *fur::gfp* remains essentially the same in the *E. coli* Δ*iscU* mutant cells grown in M9 medium supplemented with 0, 1.0 μM, 2.0 μM, and 10.0 μM iron (Fig. 3*B*), suggesting that Fur fails to repress the expression of the reporter gene *fur::gfp* in the *E. coli* mutant cells with deletion of IscU in response to elevation of intracellular free iron content.

If deletion of gene *iscU* results in Fur protein that cannot be activated in response to elevation of intracellular free iron content, it is conceivable that the *ΔiscU* mutant cells would become more sensitive to iron than wildtype cells. To test this idea, both wildtype *E. coli* and the *ΔiscU* mutant cells are grown in M9 medium supplemented with increasing concentrations of iron (0–10 μM) under aerobic growth conditions. Figure 3*C* shows that the cell growth of wildtype *E. coli* increases when M9 medium is supplemented with iron, likely because M9 medium is iron deficient (∼0.05 μM total iron) (56). In contrast, the cell growth of the *ΔiscU* mutant cells significantly decreases when M9 medium is supplemented with iron. The results further suggest that the *E. coli* mutant with deletion of gene *iscU* has an unresponsive Fur and becomes sensitive to excess iron in M9 medium.

### IscU promotes the [2Fe-2S] cluster assembly in apo-form Fur *in vitro*

For many iron–sulfur proteins, iron–sulfur clusters may be readily reconstituted with ferrous iron and sulfide in the presence of thiol-reducing agents such as dithiothreitol *in vitro* (57). However, we were unable to reconstitute the [2Fe-2S] cluster in apo-form *E. coli* Fur *in vitro*, even with excess amounts of ferrous iron and sulfide (25). Since IscU is required for the [2Fe-2S] cluster assembly in Fur in *E. coli* cells (Fig. 1), we reason that IscU may promote the [2Fe-2S] cluster assembly in *E. coli* Fur *in vitro*.

**Figure 4:**
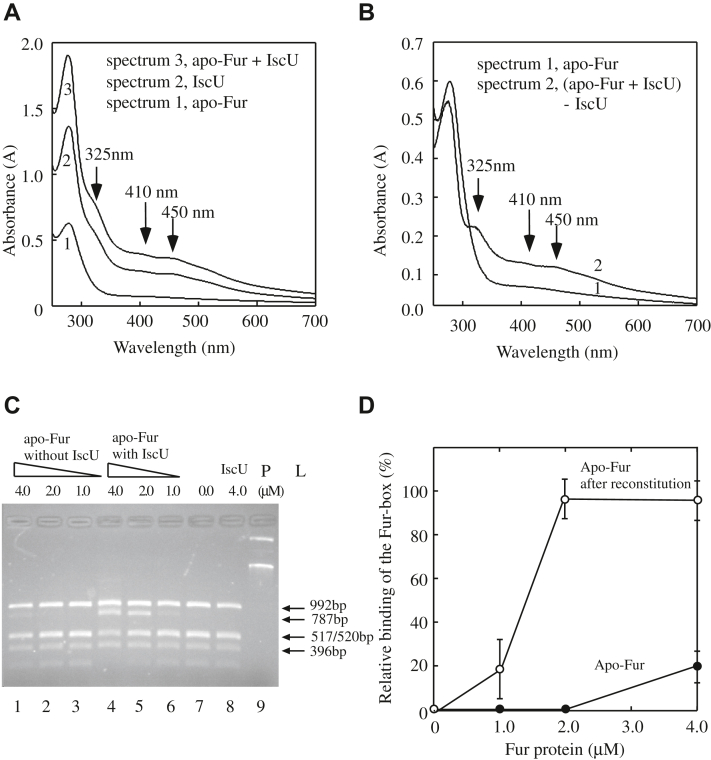
**IscU promotes the [2Fe-2S] cluster assembly in apo-form *E. coli* Fur *in vitro*.***A*, UV-Vis absorption spectra of apo-form Fur (30 μM) after reconstitution with Fe(NH_4_)_2_(SO_4_)_2_ (1.0 mM), L-cysteine (1.0 mM), cysteine desulfurase (IscS) (1.0 μM), and dithiothreitol (4 mM) in the absence (spectrum 1) or presence (spectrum 3) of IscU (50 μM) at 37 ^°^C for 20 min. Spectrum 2, IscU (50 μM) after reconstitution with Fe(NH_4_)_2_(SO_4_)_2_ (1.0 mM), L-cysteine (1.0 mM), cysteine desulfurase (IscS) (1.0 μM), and dithiothreitol (4 mM) at 37 ^°^C for 20 min. *B*, the [2Fe-2S] cluster is reconstituted in apo-form *E. coli* Fur by IscU. Spectrum 1, apo-form Fur (30 μM) after the reconstitution without IscU. Spectrum 2, the net spectrum of apo-form Fur after the reconstitution with IscU [the reconstituted IscU (spectrum 2) was subtracted from the reconstituted apo-form Fur and IscU (spectrum 3) in (*A*)]. *C*, the restriction site protection assays of apo-form *E. coli* Fur after reconstitution with or without IscU. pUC19-*iuc* was preincubated with increasing concentrations of Fur proteins, followed by digestion with *Hin*fI at 37 ^°^C for 10 min. The digested DNA products were separated on 1.5% agarose gel electrophoresis. Lanes 1 to 3, pUC19-*iuc* (3.2 nM) was preincubated with 4.0 μM, 2.0 μM, and 1.0 μM apo-form Fur after reconstitution without IscU, respectively. Lanes 4 to 6, pUC19-*iuc* (3.2 nM) was preincubated with 4.0 μM, 2.0 μM, and 1.0 μM apo-form Fur after reconstitution with IscU, respectively. Lane 7, no Fur protein was added before the *Hin*fI digestion. Lane 8, pUC19-*iuc* (3.2 nM) was preincubated with IscU (4.0 μM). Lane 9, pUC19-*iuc* only. The data are representative from three independent experiments. *D*, relative binding activity of apo-form Fur after reconstitution with or without IscU. The intensities of the DNA band of 787 bp in the agarose gel shown in (*C*) were quantified using ImageJ and plotted as a function of the Fur concentrations. Data represent mean ± SD (standard deviation) from three independent experiments. Fur, ferric uptake regulator.

Apo-form Fur (30 μM) (purified from the *E. coli* Δ*iscU* mutant cells) is incubated with ferrous ammonium sulfate (1 mM), L-cysteine (1 mM), cysteine desulfurase (IscS) (1 μM), and dithiothreitol (4 mM), with or without IscU (50 μM) at 37 ^°^C for 20 min in a cuvette. After incubation, the samples are subjected to UV-Vis absorption measurements. Figure 4*A* shows that without IscU in the reconstitution solution, very little or no [2Fe-2S] cluster is assembled in apo-form Fur (Spectrum 1), as reported previously (25). However, when IscU is included in the reconstitution solution, the [2Fe-2S] clusters are assembled in apo-form Fur (spectrum 3). Attempts to re-purify Fur from the reconstitution solution were not successful as IscU and Fur have similar molecular weights (15.3 kDa and 16.8 kDa, respectively) and similar elusion profiles from the Mono-S column (GE HealthCare co) (data not shown). While addition of a new tag is possible for repurification of Fur from the reaction solution, tags such as glutathione-S-transferase (26 kDa) or maltose binding protein (42 kDa) are too big relative to Fur and IscU and could potentially interfere with the activity of IscU and Fur. Thus, we digitally subtracted the spectrum of IscU after the reconstitution (spectrum 2) from the spectrum of apo-form Fur and IscU after the reconstitution (spectrum 3). Figure 4*B* shows the UV-Vis absorption spectra of apo-form Fur after the reconstitution without IscU (spectrum 1) and with IscU (spectrum 2), indicating that IscU promotes the [2Fe-2S] cluster assembly in apo-form Fur in the presence of ferrous iron, L-cysteine, and IscS *in vitro*.

Importantly, while apo-form Fur after the reconstitution without IscU remains inactive to bind the Fur-box (lanes 1–3), apo-form Fur after the reconstitution with IscU becomes active to bind the Fur-box (lanes 4–6). As a control, IscU alone has no Fur-box binding activity (Fig. 4*C*, lane 8). The intensities of the DNA band of 787 bp on the agarose gels are quantified using ImageJ (NIH) and plotted as a function of the Fur concentrations (Fig. 4*D*). Taken together, the results suggest that the Fur-box binding activity of Fur is restored after the [2Fe-2S] cluster is reconstituted in apo-form Fur with IscU *in vitro*.

### IscU is also required for the [2Fe-2S] cluster assembly in the *H. influenzae* Fur, but not for the [2Fe-2S] cluster assembly in the *E. coli* ferredoxin and FhuF in *E. coli* cells

**Figure 5:**
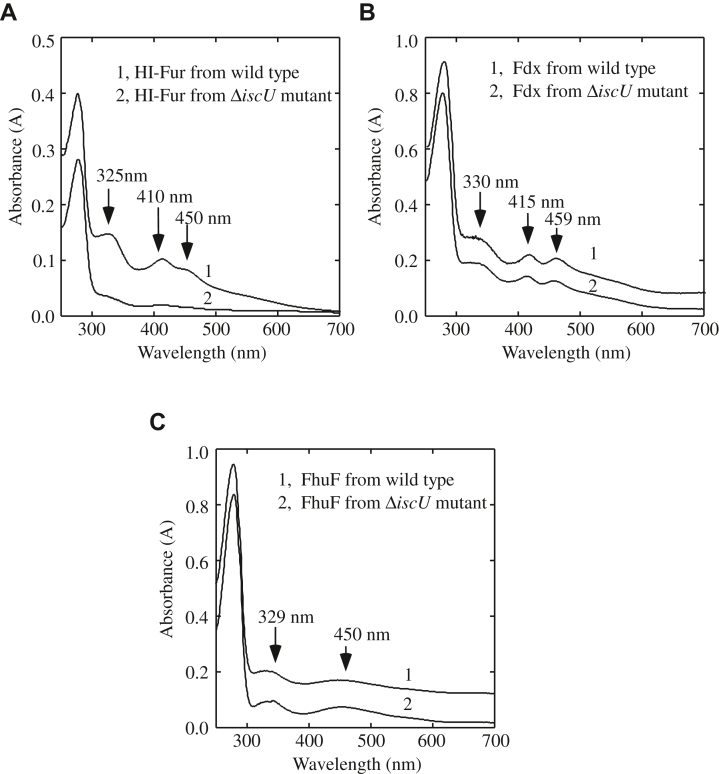
**IscU is also required for the [2Fe-2S] cluster assembly in the *H. influenzae* Fur but not for the [2Fe-2S] cluster assembly in the *E. coli* ferredoxin and FhuF in *E. coli* cells.***A*, UV-Vis absorption spectra of the *H. influenzae* Fur purified from wildtype *E. coli* (spectrum 1) and the Δ*iscU* mutant (spectrum 2) cells grown in M9 medium supplemented with 2 μM Fe(NH_4_)_2_(SO_4_)_2_ under aerobic growth conditions. Purified *H. influenzae* Fur (40 μM) was dissolved in buffer containing NaCl (500 mM) and Tris (20 mM, pH 8.0). *B*, UV-Vis absorption spectra of the *E. coli* ferredoxin (Fdx) purified from wildtype *E. coli* (spectrum 1) and the Δ*iscU* mutant (spectrum 2) cells grown in M9 medium supplemented with 2.0 μM Fe(NH_4_)_2_(SO_4_)_2_ under aerobic growth conditions. Purified ferredoxin (75 μM) was dissolved in buffer containing NaCl (500 mM) and Tris (20 mM, pH 8.0). *C*, UV-Vis absorption spectra of the *E. coli* FhuF purified from wildtype *E. coli* (spectrum 1) and the Δ*iscU* mutant (spectrum 2) cells grown in M9 medium supplemented with 2.0 μM Fe(NH_4_)_2_(SO_4_)_2_ under aerobic growth conditions. Purified FhuF (12 μM) was dissolved in buffer containing NaCl (500 mM) and Tris (20 mM, pH 8.0). The results are representatives of three independent experiments. Fur, ferric uptake regulator.

To explore whether IscU is also involved in the [2Fe-2S] cluster assembly in other Fur proteins, we expressed the *H. influenzae* Fur (HI-Fur) in *E. coli* wildtype and the Δ*iscU* mutant cells grown in M9 medium supplemented with 2 μM iron under aerobic growth conditions. Figure 5*A* shows that while HI-Fur purified from wildtype *E. coli* cells has three absorption peaks at 325 nm, 410 nm, and 450 nm of the [2Fe-2S] cluster (Spectrum 1), the HI-Fur purified from the *E. coli* Δ*iscU* mutant cells has only very small absorption peaks at 325 nm, 410 nm, and 450 nm (spectrum 2), indicating that the [2Fe-2S] cluster assembly in HI-Fur in *E. coli* cells also requires IscU.

In parallel, the *E. coli* ferredoxin, a [2Fe-2S] cluster-containing protein involved in iron–sulfur cluster biogenesis (47, 48), is expressed in *E. coli* wildtype and the Δ*iscU* mutant cells grown in M9 medium supplemented with 2 μM iron under aerobic growth conditions. Figure 5*B* shows that the UV-Vis absorption spectra of ferredoxin purified from wildtype *E. coli* cells and the Δ*iscU* mutant cells are essentially identical. Similarly, deletion of IscU has very little or no effects on the [2Fe-2S] cluster assembly in the *E. coli* siderophore-reductase FhuF (49, 50) (Fig. 5*C*). Thus, IscU seems to have a unique role for the [2Fe-2S] cluster assembly in Fur in *E. coli* cells.

## Discussion

Here, we report that the [2Fe-2S] cluster assembly in Fur requires the iron–sulfur cluster assembly scaffold protein IscU, as deletion of gene *iscU* inhibits the [2Fe-2S] cluster assembly in Fur and prevents activation of Fur as a repressor in *E. coli* cells in response to elevation of intracellular free iron content. The *in vitro* studies further suggest that IscU can promote the [2Fe-2S] cluster assembly in apo-form Fur and restore its Fur-box binding activity. The results demonstrate that the [2Fe-2S] cluster in Fur is enzymatically assembled by the iron–sulfur cluster assembly system and that IscU has a crucial role for activation of Fur in *E. coli* cells.

In eukaryotes, iron–sulfur cluster binding to key transcriptional or posttranscriptional regulators mediates intracellular iron sensing. In yeast, paralogs Aft1 and Aft2 activate the iron regulon when iron levels are low (58, 59). When iron levels are high, [2Fe-2S] cluster bound Aft1/2 is exported from the nucleus, and the iron regulon is no longer activated (60). In animals, cellular iron homeostasis is regulated post-transcriptionally by iron regulatory protein IRP1 (61, 62). When iron is scarce, IRP1 binds to stem-loop structures on mRNA called iron responsive elements to modulate the translation of iron homeostatic genes. When iron is replete, IRP1 binds a [4Fe-4S] cluster and functions as a cytosolic aconitase rather than an IRP (61, 62). Our work extends the paradigm of sensing iron through iron–sulfur clusters to bacteria, where we previously demonstrated that Fur binds a [2Fe-2S] cluster, not mononuclear ferrous iron as was commonly thought (1, 2, 8, 9). Moreover, we now also demonstrate that IscU, but not other iron–sulfur cluster assembly proteins like IscA or SufA, is required for [2Fe-2S] cluster assembly in Fur and that IscU plays a privileged role in activating Fur given that other iron–sulfur proteins like FhuF are not dependent on IscU (Fig. 5).

IscU has been shown to interact with cysteine desulfurase IscS and deliver the assembled iron–sulfur cluster to target proteins (37, 38, 39, 40). In eukaryotic cells, deletion of IscU leads to accumulation of iron in mitochondria (63). Using the membrane-permeable iron indicator 2,2′-dipyirdyl, we have also found that deletion of IscU elevates the chelatable intracellular iron content in *E. coli* cells grown in LB medium under aerobic growth conditions (Supplemental Materials). However, unlike the *E. coli ΔiscA*/*ΔsufA* mutant cells which also have an elevated intracellular free iron content (25), the *E. coli ΔiscU* mutant cells fail to assemble the [2Fe-2S] cluster in Fur in *E. coli* cells in response to elevation of intracellular free iron content (Fig. 1*B*). The results strongly suggest that the iron–sulfur cluster assembly proteins IscA and IscU have their distinct roles in iron–sulfur cluster biogenesis (64). In this context, we propose that while deletion IscA and its paralog SufA increases intracellular free iron content and promotes the [2Fe-2S] cluster assembly in Fur (25), deletion of IscU interrupts the [2Fe-2S] cluster assembly process in Fur and results in apo-form Fur in *E. coli* cells. It should be pointed out that IscU is not required for the [2Fe-2S] cluster assembly in ferredoxin and FhuF in *E. coli* cells grown in M9 medium supplemented with 2 μM iron, suggesting that IscU may have a unique role for the [2Fe-2S] cluster assembly in the iron regulatory protein Fur. The underlying mechanism remains to be further investigated.

**Figure 6:**
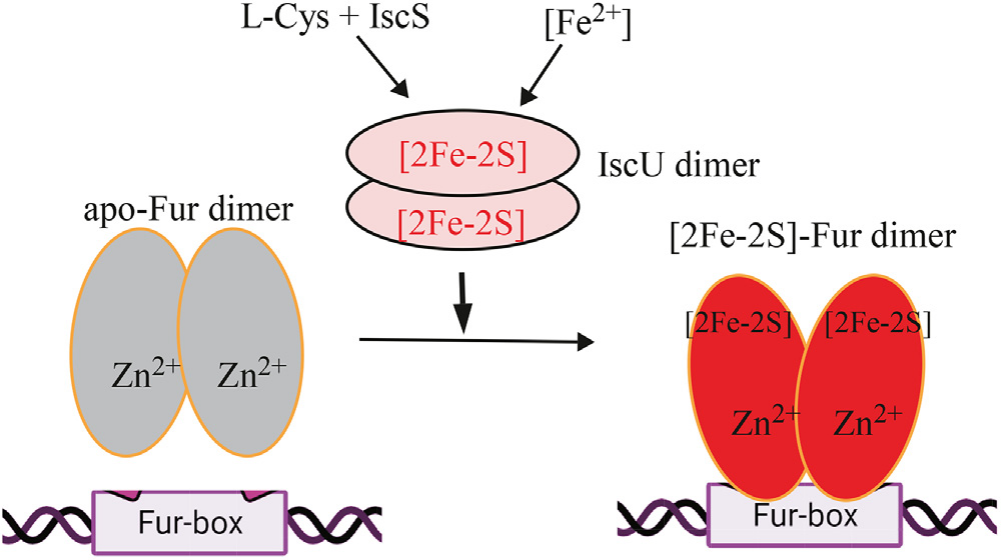
**A proposed model for the interplay between iron–sulfur cluster biogenesis and regulation of intracellular iron homeostasis in *E. coli* cells.** When intracellular free iron content is depleted, IscU cannot assemble a [2Fe-2S] cluster in Fur, and Fur is inactive. When intracellular free iron content is elevated, IscU, together with L-cysteine, cysteine desulfurase (IscS) and other iron–sulfur cluster assembly machinery, assembles a [2Fe-2S] cluster in Fur, and Fur becomes an active repressor to bind the Fur-box and regulates intracellular iron homeostasis. A structural zinc binding site is shown in apo-Fur and the [2Fe-2S] cluster-bound Fur. Fur, ferric uptake regulator.

Ironically, the expression of iron–sulfur cluster assembly systems encoded by the housekeeping *isc* operon (*iscSUA-hscBA-fdx*) (28) and the alternative *suf* operon (*sufABCDSE*) (29) is also regulated by Fur in *E. coli* cells. First, an active Fur will bind the Fur-box in the promoter of the *suf* operon and repress the expression of the operon (30, 31). Under iron starvation (30) or oxidative stress conditions (31, 32), Fur becomes inactive and dissociates from the Fur-box site, and the *suf* operon is expressed for iron–sulfur cluster biogenesis or repair (30, 31). Second, Fur indirectly regulates the expression of the *isc* operon *via* an iron-responsive RNA, RyhB (65, 66). RyhB is a small RNA (containing 90 nt) that regulates at least 18 operons and over 56 genes in *E. coli* (66). The expression of RyhB is repressed by an active Fur (65). When Fur is inactivated, highly expressed RyhB binds to specific mRNA of the *isc* operon, resulting in inhibition of iron–sulfur cluster assembly in cells (67). Our finding that the [2Fe-2S] cluster in Fur is assembled by IscU reveals a novel link between iron–sulfur cluster biogenesis and regulation of intracellular iron homeostasis in *E. coli* cells (Fig. 6).

Compared with other [2Fe-2S] proteins, *E. coli* Fur appears to have a relatively weak binding affinity for the cluster (25). For example, when the *E. coli* ferredoxin is expressed in wildtype *E. coli* cells grown in LB medium, about 35% of ferredoxin binds a [2Fe-2S] cluster (47). However, when *E. coli* Fur is expressed in wildtype *E. coli* cells grown in LB medium, only about 8% of Fur binds a [2Fe-2S] cluster (25). Furthermore, unlike many iron–sulfur proteins, the [2Fe-2S] cluster could not be readily reconstituted in apo-form *E. coli* Fur *in vitro* even with excess amounts of ferrous iron and sulfide (25). Only when IscU is included in the reconstitution solution, the [2Fe-2S] cluster is assembled in apo-form Fur in the presence of iron and L-cysteine and IscS (Fig. 4). The weak binding activity of Fur for the [2Fe-2S] cluster may reflect its regulatory nature as a sensor of intracellular free iron content. When intracellular free iron content is depleted, Fur does not bind the [2Fe-2S] cluster and is inactive. When intracellular free iron content is elevated, the [2Fe-2S] cluster is enzymatically assembled in Fur, and Fur becomes active to repress the expression of iron uptake systems, the iron-responsive RNA RyhB, and many other target genes (65, 66). Repression of RyhB expression will then enhance the expression of the *isc* operon and promote iron–sulfur cluster biogenesis activity in *E. coli* cells (67). Thus, regulation of intracellular iron homeostasis and iron–sulfur cluster biogenesis are closely coupled *via* this global iron regulator Fur in *E. coli* cells.

## Experimental procedures

### Protein purification

Gene *iscU* in wildtype *E. coli* cells (MC4100) was deleted in-frame using the single-step inactivation procedure (52) as described previously (43). Deletion of *iscU* was confirmed by PCR using two primers flanking the *iscU* gene. Plasmid pBAD expressing native *E. coli* Fur (25), his-tagged *H. influenzae* Fur (25), his-tagged *E. coli* ferredoxin (47), or his-tagged FhuF (49, 50) was introduced into wildtype and the *ΔiscU* mutant *E. coli* cells as described previously (25). Overnight *E. coli* cultures were inoculated 1:100 dilution in freshly prepared M9 medium supplemented with 20 amino acids (10 μg each amino acid/ml), thiamine (1 μg/ml), glycerol (0.4%), ampicillin (100 μg/ml), and Fe(NH_4_)_2_(SO_4_)_2_ (0–10.0 μM). When the cells were grown to *A* at 600 nm of ∼0.6 at 37 ^°^C under aerobic growth condition, the protein expression was induced by adding L-arabinose (0.04%). The cells were grown for 3 more hours, and Fur was purified from the cells as described previously (25). The purity of purified Fur proteins was more than 90% as judged from the electrophoresis analysis on a 15% polyacrylamide gel containing SDS followed by staining with Coomassie Blue. The concentration of purified protein was measured at 280 nm after iron–sulfur clusters in protein were removed by adding HCl (10 mM). The extinction coefficients of 5.6 mM^−1^ cm^−1^, 6.9 mM^−1^ cm^−1^, 9.2 mM^−1^ cm^−1^, 56.4 mM^−1^ cm^−1^, 42 mM^−1^ cm^−1^, and 11.2 mM^−1^ cm^−1^ at 280 nm were used for calculating the concentration of apo-form *E. coli* Fur, apo-form *H. influenzae* Fur, apo-form *E. coli* ferredoxin, apo-form *E. coli* FhuF, *E. coli* IscS, and *E. coli* IscU, respectively. The concentration of the [2Fe-2S] cluster in Fur was determined using an extinction coefficient of 10 mM^−1^ cm^−1^ at 410 nm (25). The occupancy of the [2Fe-2S] cluster in Fur was calculated from the ratio of the [2Fe-2S] cluster to Fur monomer.

### *Hin*fI site protection assays

The Fur-box binding activity of *E. coli* Fur was analyzed using the *hin*fI site protection assays (9). The Fur-box in the *E. coli iucABCD* promoter (5′-GA**GAATC**ATTAGCATTCGC-3′) contains the restriction enzyme *Hin*fI site (5′-GAATC-3′). Binding of Fur to the Fur-box protects the *hin*fI site (highlighted) from being cleaved by *Hin*fI (9). The *iucABCD* promoter was synthesized (GenScript co.) and cloned into plasmid pUC19 *via Bam*HI and *Hin*dIII sites to create pUC19-*iuc*. For the *hin*fI site protection assays, pUC19-*iuc* was preincubated with purified Fur proteins in 10 μl reaction solutions containing MgCl_2_ (2 mM), NaCl (150 mM), bovine serum albumin (0.1 mg/ml), and Tris (20 mM, pH 8.0) for 10 min at room temperature. Restriction enzyme *Hin*fI (0.5 unit) (New England Biolab co.) was then added to the reaction solutions. After incubation at 37 ^°^C for 10 min, the reaction was stopped by adding 2 μl 6× loading buffer (New England Biolab co). The digested DNA products were separated on 1.5% agarose electrophoresis gel containing ethidium bromide (0.1 μg/ml) in 0.5× TAE (Tris-acetate-EDTA) buffer, run at 120 V for 35 min. The gel images were taken using the Kodak Gel Logic 200 Imaging System. The intensities of the DNA band on the agarose gels were quantified using ImageJ (NIH).

### *In vivo* repressor activity assays of Fur using the reporter gene *Fur::gfp*

A reporter gene *fur::gfp* with the *E. coli fur* promoter: (5′-ATGTCTACGCCGTATTAATAGATAATGCCAATCAAAATAATTGCTACAAATTTGTAACTTTTGCTGTTGTACCTGTACAATGTCCCGGTGTTCAAGTGGCCTTGCCGTTGTAAATGTAAGCTGTGCCACGTTTTTATTAACAATATTTGCCAGGGACTTGTGGTTTTCATTTAGGCGTGGCAATTC**TATAATGATACGCATTATC**TCAAGAGCAAATTCTGTCACTTCTTCTAATGAAGTGAACCGCTTAGTAACAGGACAGATTCCGC-3′) and the *gfp* gene (Addgene co) was synthesized (GenScript co) and cloned into plasmid pBAD *via EcoR*V and *Hind*III sites. The highlighted sequence represents the Fur-box (10, 11). The plasmid containing the reporter gene *fur::gfp* was introduced into wildtype *E. coli* (MC4100) and the Δ*iscU* mutant cells. Overnight culture was inoculated (1:100 dilution) in freshly prepared M9 medium supplemented with 20 amino acids (10 μg each amino acid/ml), thiamine (1 μg/ml), glycerol (0.4%), Fe(NH_4_)_2_(SO_4_)_2_ (0–10.0 μM), and ampicillin (100 μg/ml), and grown at 37 ^°^C under aerobic condition for 5 h. Cells were then subjected to the GFP fluorescence measurements at 507 nm using the excitation wavelength at 481 nm in the fluorescence spectrometer (PerkinElmer LS-3). The relative fluorescence intensity was calculated by dividing the green fluorescence intensity with the cell density (*A* at 600 nm). The *E. coli* cells without the reporter gene *fur::gfp* grown in M9 medium supplemented with increasing concentrations of Fe(NH_4_)_2_(SO_4_)_2_ (0–10.0 μM) were used as the controls.

### *In vitro* reconstitution of the [2Fe-2S] cluster in apo-form *E. coli* Fur

Apo-form *E. coli* Fur (purified from the *E. coli* Δ*iscU* mutant cells) was used for the iron–sulfur cluster reconstitution *in vitro*. Apo-form Fur (30 μM) was incubated with Fe(NH_4_)_2_(SO_4_)_2_ (1 mM), L-cysteine (1 mM), cysteine desulfurase (IscS) (1 μM), and dithiothreitol (4 mM) with or without *E. coli* IscU (50 μM) in a cuvette at 37 ^°^C. After 20 min incubation, UV-Vis absorption spectra of the samples were taken in a Jasco V-750 UV-Vis absorption spectrometer equipped with a temperature controller. For the baseline, IscU (50 μM) was incubated with Fe(NH_4_)_2_(SO_4_)_2_ (1 mM), L-cysteine (1 mM), cysteine desulfurase (IscS) (1 μM), and dithiothreitol (4 mM) at 37 ^°^C for 20 min. The net spectrum of the IscU-mediated reconstitution in Fur was obtained by subtracting the spectrum of the reconstituted IscU from the spectrum of the reconstituted apo-form Fur and IscU.

### Statistical analysis

All data are expressed as mean ± SD (standard deviation) from at least three independent experiments.

## Data availability

All data generated and analyzed in the present study are included in the manuscript. Raw data are available on request.

## Supporting information

This article contains supporting information (68).

## Conflict of interest

The authors declare that they have no conflicts of interest with the contents of this article.
