# Supplementary material for: Iron–sulfur cluster assembly scaffold protein IscU is required for activation of ferric uptake regulator (Fur) in Escherichiacoli
Source: J Biol Chem. 2024 Mar 5;300(4):107142. doi: 10.1016/j.jbc.2024.107142 (PMC11001641; doi:10.1016/j.jbc.2024.107142)
Supplement: Supplementary Figure 1 [file mmc1.pdf]

Supplementary Figure 1

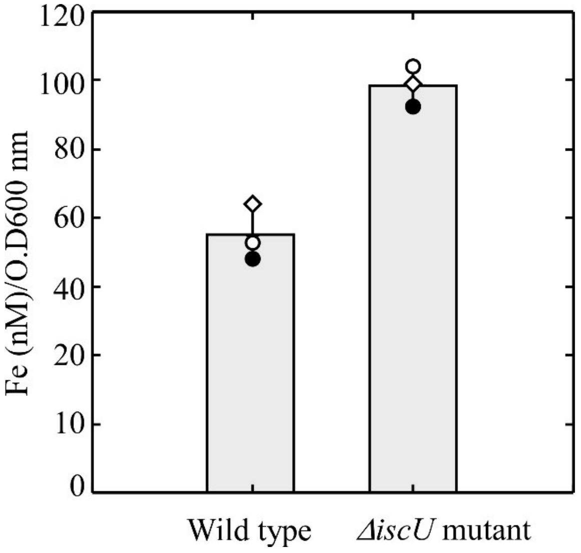

Supplementary Figure 1. Deletion of IscU elevates the intracellular chelatable iron content in *E. coli*. Overnight culture of wild type (MC4100) or the *ΔiscU* mutant was inoculated (1:100) in LB medium, grown at 37°C under aerobic growth condition for 2 hours, followed by adding a membrane permeable iron chelator 2'2-dipyridyl (50 μM). After additional 1 hour growth, cells were harvested, washed with buffer containing NaCl (500 mM) and Tris (20 mM, pH 8.0) once, and disrupted by passing through French Press. After centrifugation to remove cell debris, the supernatant of cell extract was subjected to UV-Vis absorption measurements. The cell extract without addition of 2,2'-dupyridyl was used as the control. An extinction coefficient of 9.95 $mM^{-1}cm^{-1}$ at 522 nm (1) was used to determine the concentration of the Fe^{2+}-dipyridyl complex in the cell extract. The relative intracellular chelatable iron content was calculated from the ratio of the iron concentration in the cell extract to the cell density (O.D. at 600 nm). The data represent the mean ± SD (standard deviation) of three independent experiments.

1. Smith, G. L., Reutovich, A. A., Srivastava, A. K., Reichard, R. E., Welsh, C. H., Melman, A. *et al.* (2021) Complexation of ferrous ions by ferrozine, 2,2'-bipyridine and 1,10-phenanthroline: Implication for the quantification of iron in biological systems J Inorg Biochem **220**, 111460 10.1016/j.jinorgbio.2021.111460
